# Variation in Rumen Bacteria of Lacaune Dairy Ewes From One Week to the Next

**DOI:** 10.3389/fmicb.2022.848518

**Published:** 2022-06-23

**Authors:** Solène Fresco, Christel Marie-Etancelin, Annabelle Meynadier, Guillermo Martinez Boggio

**Affiliations:** GenPhySE, INRAE, INPT, ENVT, Université de Toulouse, Castanet-Tolosan, France

**Keywords:** rumen bacteria, dairy sheep, fatty acids, compositional data, stability, repeatability

## Abstract

Bacteria are the most abundant microorganisms in the rumen microbiota and play essential roles, mainly fermenting plant compounds that yield fatty acids. In this study, we aimed at assessing stability of both bacterial composition and of its associations with rumen and milk fatty acids phenotypes over a 1-week period. The study was performed using 118 Lacaune dairy ewes from the INRAE Experimental Unit of La Fage. Rumen and milk samples were obtained from the ewes twice, 1 week apart, and microbiota composition, volatile and long-chain fatty acid concentrations were analyzed. Bacterial composition was assessed using 16S rRNA gene sequencing, and microbiota and fatty acids were analyzed as compositional data. As we worked with relative abundances expressed in a constrained space, the centered log-ratio transformation enabled to transform data to work with multivariate analyses in the Euclidian space. Bacterial composition differed between the 2 weeks of sampling, characterized by different proportions of the two main phyla, *Bacteroidetes* and *Firmicutes*. The repeatability of the operational taxonomic units (OTUs) was low, although it varied significantly. However, 66 of them presented a repeatability of over 0.50 and were particularly associated with fatty acid phenotypes. Even though the OTUs from the same bacterial families presented similar correlations to fatty acids in both weeks, only a few OTUs were conserved over the 2 weeks. We proved with the help of sequencing data that there is significant change in microbial composition over a week in terms of abundance of different families of bacteria. Further studies are required to determine the impact of bacterial composition alterations over 1 week, and the specificities of the highly repeatable OTUs.

## Introduction

Ruminant evolution resulted in adaptations to digest plant fiber efficiently through the development of multi-chamber stomachs hosting a microbiota composed of bacteria, archaea, protozoa, and fungi (Dehority, 2003). Among these organisms, bacteria are the most abundant and contribute the most to energy production, mainly volatile fatty acids (VFAs), from the fermentation of plant carbohydrates (Hungate, 1966). They are also involved in lipolysis and biohydrogenation (Hungate, 1966) and thus influence the long-chain fatty acid (LCFA) composition in the rumen, which in turn greatly determines milk LCFA composition (Lourenço et al., 2010; Jami et al., 2014; Buitenhuis et al., 2019).

The rumen bacterial composition of adults can be influenced by biological factors, such as diet (Fernando et al., 2010; Henderson et al., 2015), parity (Pitta et al., 2014), and genetics (Sasson et al., 2017; Difford et al., 2018). Technical and computational factors may induce bias in the observed bacterial composition (Wang and LêCao, 2020). Some of the main factors include sampling techniques (Geishauser and Gitzel, 1996; Lodge-Ivey et al., 2009; Henderson et al., 2013), the bioinformatics pipeline (Schloss, 2010), and the statistical approach, including normalization method and tests applied to the data (McMurdie and Holmes, 2014). A specific methodology must be applied to microbiota data which is considered as compositional data (Gloor et al., 2017), as the only relevant information is contained in the ratios between the variables, and not in their numerical values.

Microbiota stability can be defined as the conservation of bacterial proportions at different taxonomic levels over a period of time, or high repeatability of bacterial abundance. Studies have shown that during adulthood of ruminants, bacterial composition appears to vary over long periods (3–4 months; Bainbridge et al., 2016; Zhu et al., 2021), but to be stable over shorter ones (3 days to 2 weeks; Skarlupka et al., 2019; Huang et al., 2020; Mamun et al., 2020). However, few studies focused on the short period rumen bacterial stability, and small numbers of animals were used, varying from 5 to 12 for above cited studies.

Assessing whether bacterial composition is stable over short periods of time is essential to confirm that phenotypic correlations between rumen microbiota and rumen and milk fatty acids are independent from sampling time and the influence of environmental factors (i.e., diet). The objective of this study was to answer the following two questions: Is the rumen bacterial composition of dairy ewes stable over a short period? Does it affect the correlations of bacteria with rumen and milk fatty acids?

## Materials and Methods

### Animals and Experimental Design

Data were obtained from 118 Lacaune dairy ewes reared on the Experimental Unit La Fage (INRAE UE 321 agreement A312031, Roquefort, France). The genetic structure of the INRAE La Fage flock includes independent divergent genetic lines of Lacaune dairy ewes: two selected for milk SCS, based on estimated breeding values (EBVs) for milk SCS, and the other two for PERS, based on EBVs for the coefficient of variation of milk production on the testing day. Two groups of ewes with extreme EBVs were created according to the log-transformed somatic cell count (SCC): a high-SCS line (SCS+) and a low-SCS line (SCS−). And also, two extreme groups of ewes were generated, one with high persistence (PERS+) and one with low persistence (PERS-) in the milk production curve (Table 1). Ewes were in second (*n* = 67) or third lactation (*n* = 51), ranging from 119 to 133 days in milk, and were milked twice a day (8 a.m. and 5 p.m.) with an average daily production of 1.53 ± 0.30 kg. Ewes were fed with a total of 7 kg of a mixture composed of 72% grass silage, 21% hay, and 7% barley (on a DM basis) after morning and evening milkings. They were also supplied with 100 g of barley and 100 g of a commercial protein-rich concentrate (Brebitanne®, RAGT, Albi) in the milking parlor. Ewes had free access to water and stayed indoors with no access to grazing.

**Table 1:** Number of animals per genetic line and genotype.

	Genetic lines^1^			
Genotypes^2^	PERS+	PERS−	SCS+	SCS−
CC	21	25	18	23
CT	9	4	10	7
TT	0	0	1	0

1 *Genetic lines selected for high (PERS+) and low (PERS−) *milk persistency or high (SCS+) and low* (SCS−) somatic cell score*.

2 *C being the wild allele and T the mutant allele of the SOCS2 gene*.

### Samples and Data Collection

Samples were collected on 2 days that were 1 week apart, following the same protocol. The animal was immobilized standing in a restraint cage, and a medical gastric tube introduced into the esophagus until reaching the rumen, the introduction depth being standardized by a graduation on the tube. The vacuum pump was turned on once the gastric tube in the rumen and turned off before removing it. The sample was placed in a cold storage box for transport to the lab, where three aliquots were collected: (1) the first aliquot of 2 ml for microbiota analysis; (2) the second aliquot of 5 ml of rumen fluid was mixed with 0.2 ml of sulfuric acid (25% v/v) for VFA analysis; and (3) the last aliquot of 40 ml for LCFA analysis. All three aliquots were frozen and stored at −20°C until analysis, except for those used for microbiota analysis, which were stored at −80°C. The gastric tube was cleaned with hot water after each collection.

Rumen samples were collected before milking from each animal. For both days of sampling, a first group of 60 animals were collected from 8:30 to 11:30 a.m. and a second group of 60 animals were collected from 1:30 to 4:30 p.m. The same ewes were always sampled in the morning or the evening. To avoid dilution of samples by water, the ewes were denied access to water 2 h before sampling. Animals did not have access to feed from the previous evening (10:00 p.m.) for the group sampled in the morning, and from the early morning (7:00 a.m.) for the group sampled in the afternoon. Consequently, the number of hours of fasting varied from 6.5 to 13.5 depending on the order and moment of sampling.

For each of the 2 weeks, milk yields were recorded on the previous evening and the morning of the sampling day, and milk samples were collected and preserved with bronopol (Agrolab, Aurillac, France) for milk composition analysis or by immediate freezing for LCFA analysis. Somatic cell count and fat and protein contents analyses were performed on both the evening and the morning milk samples. LCFA analysis was performed on the morning milk samples only.

### Bacterial DNA Extraction, PCR Amplification, and Sequencing

Total DNA from 80 μl of rumen samples was extracted and purified using the QIAamp DNA Stool Mini Kit (Qiagen Ltd., West Sussex, United Kingdom) according to the manufacturer’s instructions, with a bead-beating step in a FastPrep instrument (MP Biomedicals, Illkirch, France). The 16S rRNA V3–V4 regions gene of the extracted DNA strands were amplified (first PCR: 30 cycles) with the primers F343 (5′-CTTT CCCTACACGACGCTCTTCCGATCTACGGRAGGCAGCAG-3′; Liu et al., 2007) and R784 (5′-GGAGTTCAGACGTGTGCTCTTCCGATC TTACCAGGGTATCTAATCCT-3; Andersson et al., 2008). As Illumina MiSeq technology enables 250 bp reads, the ends of each read were overlapped and stitched together to generate full-length reads of the entire V3 and V4 regions in a single run. Single multiplexing was performed using a 6 bp index, which was added to R784 during a second round of PCR with 12 cycles using the forward primer (AATGATACGGCGACCACCGAGATCTACACTCT TTCCCTACACGAC) and reverse primer (CAAGCAGAAGACGGCATACGAGATGTGACT GGAGTTCAGACGTGT). The PCR products were purified and loaded onto an Illumina MiSeq cartridge (Illumina, San Diego, CA, United States) at the Genomic and Transcriptomic Platform (INRAE, Toulouse, France) according to the manufacturer’s instructions.

Sequence reads were demultiplexed, and each paired-end read was assigned to its sample based on the previously integrated index, and processed with the FROGS 3.0 pipeline (Escudié et al., 2018). The procedure consisted of the following steps: (1) read pre-processing, removing sequences with primer mismatch, displaying unexpected length (<300 or >500 bp), or with ambiguous bases; (2) sequence clustering with denoising and one sequence difference between each of the three aggregation steps of clustering; (3) chimera removal; (4) cluster filtering with Bokulich filter (removing clusters with abundances <0.005%; Bokulich et al., 2013); and (5) taxonomy assignment to operational taxonomic units (OTUs) using the SILVA 138.16S pintail 100 database. From this process, an abundance table containing the number of sequences per OTU and rumen sample was obtained.

### Rumen Fatty Acids Composition Analyses

Two gas chromatography analyses were performed at the National Veterinary School of Toulouse (Toulouse, France) with rumen samples: one for VFAs and the other for LCFAs.

Six VFAs, acetic acid (C2:0), propionic acid (C3:0), butyric acid (C4:0), valeric acid (C5:0), isobutyric acid (*iso*-C4:0), and isovaleric acid (*iso*-C5:0), were quantified using automated gas separation, according to the method of Playne (1985) and modified as follows. The rumen samples were first centrifuged at 2,880 × *g* for 20 min to separate the liquid phase. For protein removal, 1 ml of supernatant was mixed with 200 μl of (25% v/v) metaphosphoric acid and further centrifuged at 20,000 × *g* for 15 min. Then, 100 μl of the supernatant was added to 75 μl (0.2% v/v) of 4-methylvaleric acid as an internal standard and 900 μl of ultrapure water. From this mixture, 1 μl was then injected into a gas chromatograph (Hewlett Packard, Model 7890A) equipped with a DB-FFAP column (30 m × 0.53 mm i.d., 1-μm film thickness, Agilent Technologies, Palo Alto, CA, United States) and an FID detector (Avondale, PA, United States). Chromatograms were integrated using Chromeleon software (Thermo Fisher Scientific, version 6.8, Waltham, MA, United States). The sum of the six VFA concentrations was defined as the total concentration and was used to obtain the molar proportions of each VFA.

The LCFAs of rumen content were extracted and methylated *in situ* using the procedure described by Park and Goins (1994), except that the solution of 14% boron trifluoride in methanol was replaced by a solution of methanol–acetylchloride (10:1). Nonadecanoic acid (C19:0) was used as the internal standard at a dose of 0.8 mg. The fatty acid methyl esters (FAMEs) were then quantified by gas chromatography (Agilent 6890N, Network GC System, equipped with a model 7,683 auto injector, Agilent Technologies, Palo Alto, CA, United States) using a fused silica capillary column (100 m × 0.25 mm i.d., 0.20 μm film thickness, CPSil 88, Varian, Middelburg, the Netherlands) as described by Zened et al. (2011). Peaks were identified and quantified by comparison with commercial standards (Sigma Co., St Louis, MO, United States), except for C18:1, C18:1 *trans*-9, C18:1 *trans*-11, and C18:1 *cis*-9, which were identified by the order of elution. Chromatograms were integrated using the Peak Simple software (Peak Simple Data System, version 2.83, SRI, Torrance, CA, United States). Results are expressed as the percentage of total FAME. The 29 measured fatty acids (FAs) were: C12:0, C13:0, *anteiso*-C13:0, *iso*-C13:0, C14:0, *iso*-C14:0, C15:0, *anteiso*-C15:0, *iso*-C15:0, C16:0, C17:0, *anteiso*-C17:0, *iso*-C17:0, C18:0, C18:1 *cis-9*, a mix of C18:1 *cis-11* and C18:1 *trans-15*, C18:1 *cis-12*, C18:1 *cis-15*, a mix of C18:1 *trans-6*, *trans-7*, *trans-8*, C18:1 *trans-9*, C18:1 *trans-10*, C18:1 *trans-11*, C18:1 *trans-12*, C18:1 *trans-16*, C18:2, C18:2 *cis-9*, *trans-11*, C18:2 *trans-11*, *cis-15*, C18:3, and C20:1.

### Milk Composition Analyses

The four milk samples from each ewe were analyzed at Agrolab (Aurillac, France). Fat and protein contents were obtained using mid-infrared spectrometry (Milk-Scan™ FT 6000 instrument, Foss, Nanterre, France) and somatic cell count was quantified using a Fossomatic cell counter (Foss, Nanterre, France), to which a log-transformation was applied to obtain the SCS (Ali and Shook, 1980). The data were translated into daily variables. Daily milk yield was obtained by summing the morning and evening milk yields. Daily protein content, fat content, and SCS were computed as the average of the morning and evening values weighted by the corresponding milk yields.

Long-chain fatty acid percentages in total FAME were measured in the morning milk for the 2 weeks by gas chromatography at the National Veterinary School of Toulouse (Toulouse, France), following the same method as for the rumen samples. The 38 measured FAs included the 29 measured in the rumen in addition to C4:0, C6:0, C7:0, C8:0, C9:0, C10:0, C11:0, C14:1, and C16:1.

### Statistical Analysis

All analyses were performed using R software (R Core Team, 2021)^1^. Statistical significance was set at *p* < 0.05.

Microbiota and fatty acids data are compositional data (Gloor et al., 2017), meaning that the information is contained in the ratios between variables, not in the values themselves, due to the restrictions imposed by either the sequencing technology or the measurement unit (percentages). Thus, we applied the compositional data approach proposed by Aitchison (1986) for the composition of OTUs and FAs. It consisted of imputing zero values in the data set to transform the counts into log-ratios using the centered log-ratio (CLR). Then, the multivariate analysis described in a previous study by Martinez Boggio et al. (2021) was applied. Two approaches were applied to impute the zero values, considering their nature. In the bacterial abundance table, as zeros referred to a probability of count, geometric Bayesian multiplicative replacement was applied (cmultRepl function from the zCompositions package); in the LCFA datasets, the zero values reflected the detection limit of the chromatograph; thus, the expectation–maximization procedure was applied (lrEM function from the zCompositions package). Then, the CLR transformation was applied to OTUs, VFA, and LCFA data (clr function from the compositions package).

Table 2 presents the models used to correct the data for fixed effects and repeated measures (random animal effect) and the number of animals considered. Ewes were removed from OTUs models when one of their samples had a low sequencing quality (<500 OTUs). Ewes missing one of the two samples were removed from rumen and milk LCFA models. Ewes presenting values of VFAs under the detection limit of the gas chromatograph were removed. The fixed effects included in the models were: genetic lines (four levels: PERS−, PERS+, SCS-, and SCS+), week of sampling (two levels: “week 1” and “week 2”), parity (two levels), SOCS2 genotype (two levels: “TT/CT” or “CC”), time after feeding (six levels of equal size), and number of sequences per sample (four levels of equal size).

**Table 2:** Models for OTUs, VFAs, long-chain fatty acids (LCFAs) in the rumen, milk yield, fat and protein contents, somatic cell score, and LCFAs in the milk.

Variables	Line	SOCS2^1^	Parity	Line *x* Parity^2^	Week	Line *x* Week^2^	Time after feeding	Number of sequences	Number of ewes
**Rumen**									
OTUs	■		■	■	■		■	■	111
VFAs	♦				♦		♦		116
LCFAs	■	■			■	■			116
**Milk**									
LCFAs	♦	♦			♦	♦			117
Milk yield	♦	♦			♦				118
Fat content		♦	♦		♦				118
Protein content	♦				♦				118
Somatic cell score	♦	♦	♦						118

1 *Genotype for the SOCS2 gene*.

2 *x* *Represents the interaction between effects*.

■ *Fixed effects selected while significant for at least 10% of the variable.*

♦ *Fixed effects selected when significant for each variable*.

The significance of the fixed effects was assessed using the ANOVA function from the sasLM package. For milk yield, fat and protein contents, SCS, and the six VFAs, the fixed effects were included in the model when they were significant. For OTUs, rumen, and milk LCFAs, fixed effects were retained in the models when they were significant for at least 10% of the variables. Linear mixed models were defined for each trait (one OTU being one trait), using the lmer function from the lmerTest package, and the corresponding variances of random animal and residual effects were obtained (VarCorr function from the lme4 package), allowing to compute the repeatability, defined as the animal variance divided by the total variance. Then, Spearman correlations were performed between the OTU repeatability values and their percentage of zeros or average abundance.

To perform multivariate analyses assessing the effect of the week, residuals of OTUs and FAs obtained from ANOVA were used without correcting for the week effect (Table 2). Sparse partial least square discriminant analyses (sPLS-DA) and sparse partial least square analyses (sPLS) were performed, to assess the influence of the week on bacterial composition and identify relationships between the OTUs and the FAs, respectively (spls and splsda functions from the mixOmics package). The sparse procedure allowed to reduce the number of components, i.e., the dimensionality of the analyses, and select only the most relevant variables, i.e., the OTUs, for each component. The number of components was previously chosen to explain 90% of the variance, using a principal component analysis. The number of variables retained by component was determined using the CLR lasso penalty method considering the penalization obtained with a 10-fold cross-validation (cv.glmnet and glmnet functions from the glmnet package).

The predictive ability of the sPLS-DA model was evaluated through the overall misclassification error rate after a 5-fold cross-validation repeated 10 times (perf function). For each sPLS, the 20 OTUs presenting the highest association with the FAs were selected and Pearson correlations were computed. Only the FAs common in both weeks and having at least one significant correlation with the 20 selected OTUs were presented. Fisher exact tests were performed to estimate over- or under-representation of specific phyla or highly repeatable OTUs among those selected for sPLS-DA and sPLS compared to all OTUs.

## Results

### Rumen Bacterial Composition

A total of 2,500,763 sequences were obtained, with an average of 10,736 ± 3,745 reads per sample, grouped into 2,079 OTUs, with an average of 1,406 ± 211 OTUs per sample. Ten phyla were identified; the three major phyla, based on the percentage of sequences assigned to each phylum out of the total number of DNA sequences, were *Bacteroidetes* (53.5%), *Firmicutes* (35.4%), and *Proteobacteria* (6.2%). The other phyla were *Fibrobacterota* (2.7%), *Spirochaetota* (1.2%), *Actinobacteria* (0.5%), *Patescibacteria* (0.3%), and *Desulfabacterota*, *Elusmicrobiota*, and *Campylobacterota*, each lower than 0.2%. We found that 81.2% of the DNA sequences enabled genus-level classification, resulting in the identification of 117 genera. The most important ones were *Prevotella* (30.78%), *Lachnospiraceae*_*NK3A20_group* (6.3%), *Ruminococcus* (5.3%), *Christensenellaceae*_*R_7*_*group* (4.7%), *Sphingomonas* (4.5%), and *Rikenellaceae_RC9_gut_group* (3.9%). The lowest taxonomic rank considered in this study was genus, as 92.7% of the reads were assigned to unknown species or were multi-affiliated species as expected with short 16S reads.

### Fixed Effects and Repeatability

Operational taxonomic unit data were corrected by six effects that were significant for at least 10% of the OTUs: genetic lines (25.9%), week of sampling (16.2%), time after feeding (13.9%), number of sequences per sample (12.8%), parity (11.4%), and the interaction between the genetic line and parity (11.9%), with percentages in brackets indicating the percentage of OTUs for which the given factor was significant. The explained variance was estimated for all OTUs, resulting in an average of 0.12 ± 0.04, ranging from 0.03 to 0.50 according to OTUs.

**Figure 1:**
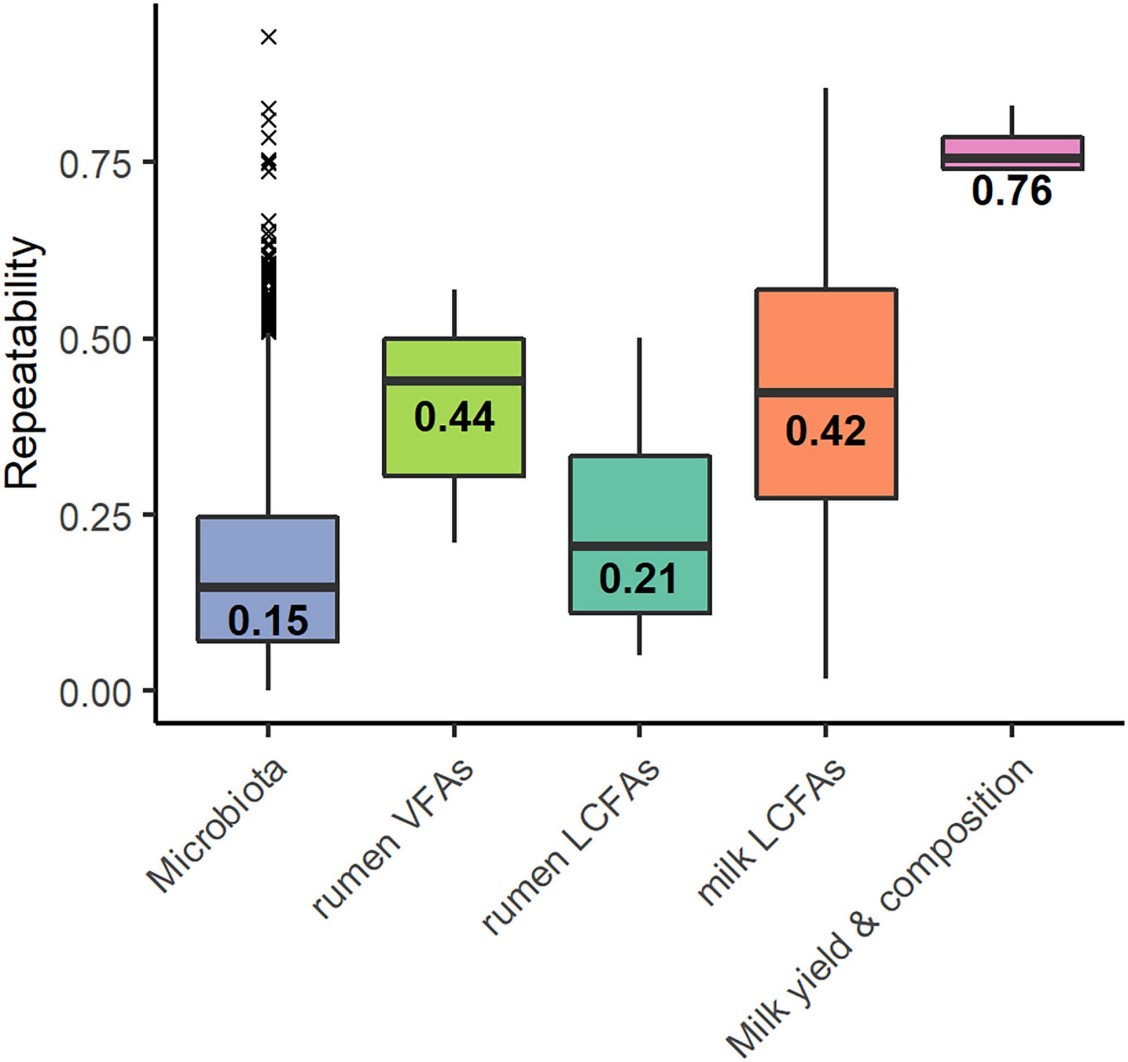
Repeatability values of the 1,599 operational taxonomic units (OTUs)* of the microbiota, the six volatile fatty acids (rumen VFAs), the 27 rumen long-chain fatty acids* (rumen LCFAs), the 37 milk long-chain fatty acids* (milk LCFAs) and the traits milk yield, and fat content and protein content and somatic cell score (Milk yield & composition). Values indicate the median. *Repeatability values were estimated for the OTU and trait models that converged.

Out of the 2,079 OTUs, 1,665 showed an estimated repeatability ranging from 0 to 0.93 with a median of 0.15 ± 0.14 (Figure 1). The remaining 414 OTUs had no estimate; linear models did not converge because of the small number of samples. Of the 1,665 OTUs, 66 presented a repeatability of over 0.50, corresponding to a frequency of 4%. Spearman correlations were performed between the repeatability values of the OTUs and their percentage of zeros (*R* = −0.01, *p* = 0.50) and between the repeatability of the OTUs and their average abundance (*R* = 0.24, *p* < 0.001).

The repeatabilities ranged from 0.21 to 0.57 for VFAs, from 0.05 to 0.50 for rumen LCFAs, and from 0.02 to 0.86 for milk LCFAs (Figure 1). The repeatability was 0.83 for milk yield, 0.74 for fat content, 0.77 for protein content, and 0.74 for SCS. The models did not converge for the LCFAs C18:1 *trans*-12 and C18:2 *cis*-9, *trans*-11 in the rumen and C20:1 in the milk.

### Bacterial Composition Over One Week

**Figure 2:**
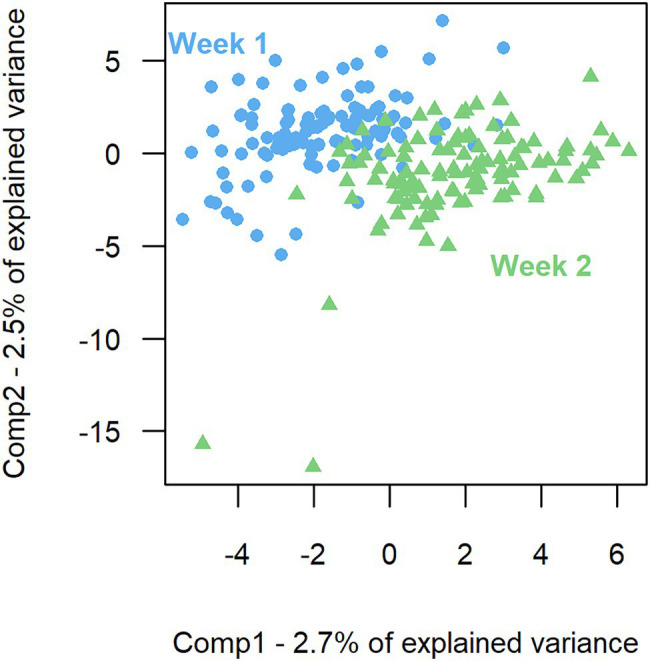
Sparse partial least square discriminant analysis of the rumen bacterial composition between the 2 weeks of sampling. First component (Comp1) plotted against second component (Comp2) presenting the largest explained variance between weeks.

The model of the sPLS-DA on bacterial composition over a week included 160 components and 57 variables per component. Based on OTU residual abundances, rumen samples were discriminated by the week of collection (Figure 2). The overall error rate of the model was 0.21. In the first component (Table 3), week 2 was characterized by a lower number of OTUs belonging to the phylum *Firmicutes* (*p* < 0.001) and a higher number of OTUs belonging to the phylum *Bacteroidetes* (*p* < 0.01) than when considering all OTUs. In the second component (Table 3), week 1 had a higher presence of OTUs from the phyla *Firmicutes* (*p* < 0.01) and a lower presence of OTUs from the phyla *Bacteroidetes* (*p* = 0.01) than when considering all OTUs.

**Table 3:** Number of OTUs belonging to the phyla *Firmicutes* and *Bacteroidetes* considering the 2,079 OTUs or the OTUs selected on the two main components for the sPLS-DA associated with each of the 2 weeks.

	Total number of OTUs	Number of OTUs from the phyla *Firmicutes*	Number of OTUs from the phyla *Bacteroidetes*
All OTUs	2,079	880	1,026
Component 1			
week 1	25	12	8
week 2	32	4	24
Component 2			
week 1	32	6	23
week 2	25	8	15

In the first component, 49 OTUs among the 57 selected to discriminate week 1 and week 2 had a repeatability of less than 0.50 (Supplementary Table S1), three had a repeatability of over 0.50, and the remaining five OTUs did not have an estimated repeatability. In the second component (Supplementary Table S2), 44 OTUs of 57 had a repeatability of less than 0.50, one had a repeatability of over 0.50, and the remaining 12 OTUs had no estimated repeatability. The proportion of OTUs presenting repeatability values of less than 0.50 among the 57 selected for each component of the sPLS-DA was not significantly different from that considering all OTUs (*p* = 0.46 and *p* = 0.47 for components 1 and 2, respectively).

### Correlations Between Rumen Bacteria and Fatty Acid Phenotypes

#### Correlations Between Rumen Bacteria and VFAs

**Figure 3:**
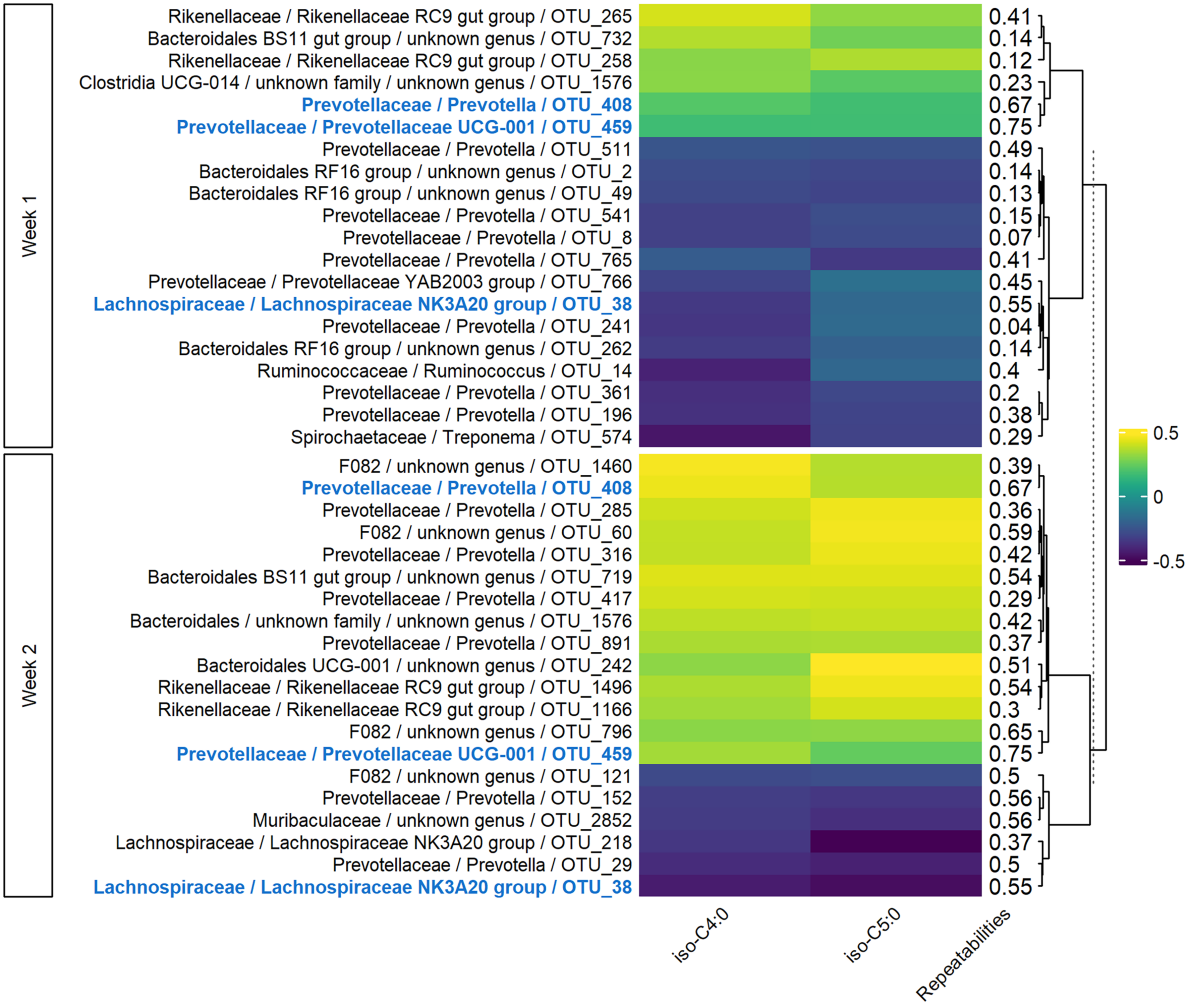
Pearson correlations between bacterial OTUs and VFAs on both weeks of sampling. Only the 20 more correlated OTUs and VFAs having at least one significant correlation in both weeks with them were presented. In bold and blue, OTUs conserved from week 1 to week 2.

In both weeks 1 and 2, sPLS models for VFAs included 77 components, with 152 variables retained per component in week 1 and 187 in week 2. For the two main components, the explained variance was 7 and 9% for weeks 1 and 2, respectively. The significant Pearson correlations between the 20 most associated OTUs and *iso*-C4:0 and *iso*-C5:0 ranged from −0.49 to −0.19 and from 0.21 to 0.46 (Figure 3). Three OTUs were conserved between week 1 and week 2, and the corresponding correlations were of the same sign. For *Lachnospiraceae* and *Rikenellaceae* families, the sign of the correlation with a given VFA was the same for all the OTUs of the family on both weeks, while bacteria from *Prevotellaceae* family had both positive and negative correlations with the VFAs. Highly repeatable OTUs (> 0.50) were particularly selected by the sPLS on both weeks (*p* = 0.04 and *p* < 0.001 for weeks 1 and 2, respectively).

#### Correlations Between Rumen Bacteria and Rumen LCFAs

**Figure 4:**
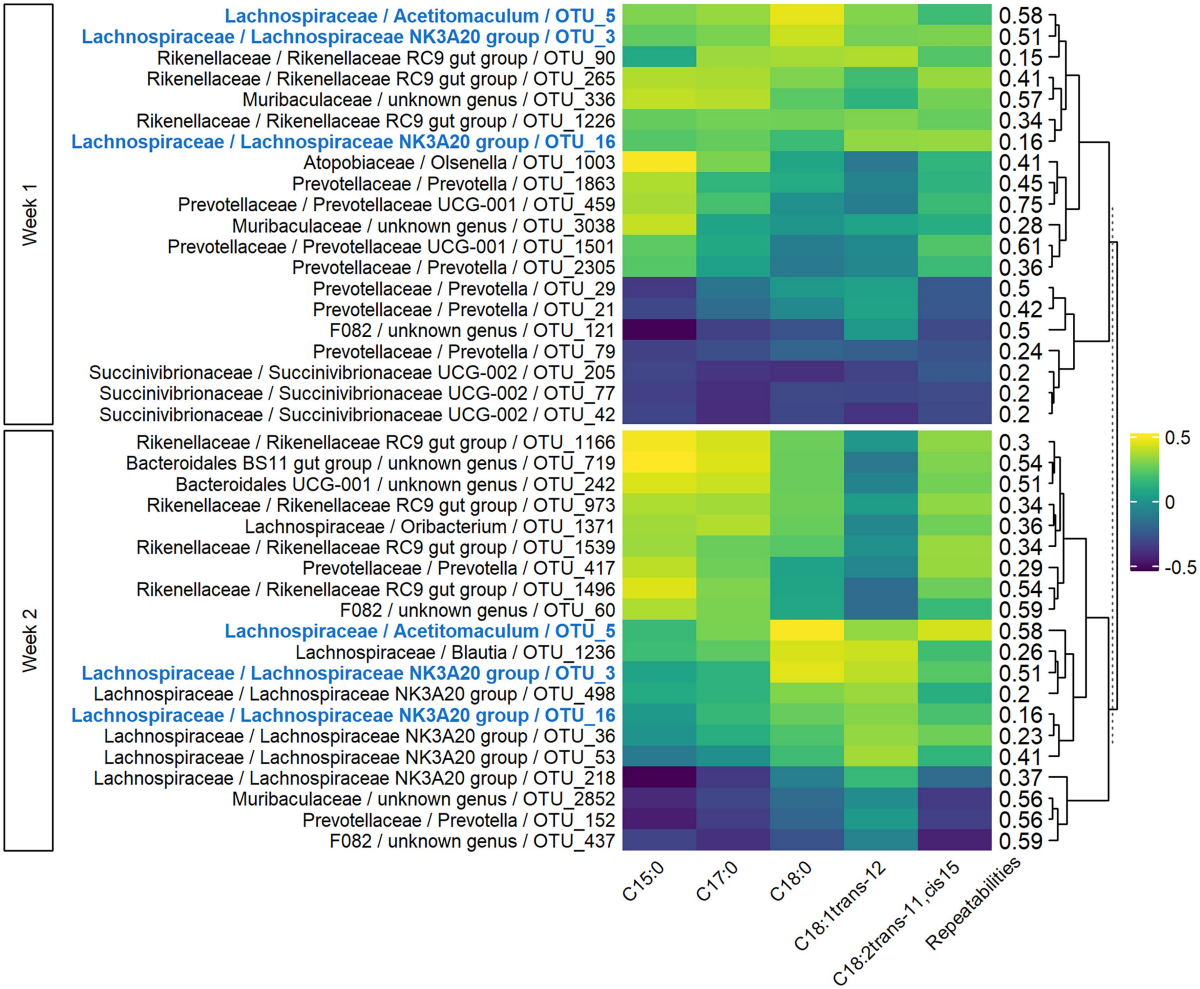
Pearson correlations between bacterial OTUs and rumen long-chain fatty acids on both weeks of sampling. Only the 20 more correlated OTUs and rumen LCFAs having at least one significant correlation in both weeks with them were presented. In bold and blue, OTUs conserved from week 1 to week 2.

For rumen LCFAs, sPLS models in both week 1 and week 2 included 77 components, with 19 variables retained per component on week 1 and 15 on week 2. For the two main components, the explained variance was 7 and 8% for weeks 1 and 2, respectively. The significant Pearson correlations ranged from −0.51 to −0.19 and from 0.19 to 0.48 for the 20 OTUs most associated with the five rumen LCFAs (Figure 4). Three OTUs appeared on both weeks, and the sign of their correlation was conserved. For *Lachnospiraceae* and *Rikenellaceae* families, the sign of the correlation with a given rumen LCFA was the same for all the OTUs of the family on both weeks, while bacteria from *Prevotellaceae* family had both positive and negative correlations with the rumen LCFAs. Highly repeatable OTUs (>0.50) were particularly selected by the sPLS on both weeks (*p* < 0.001).

#### Correlations Between Rumen Bacteria and Milk LCFAs

**Figure 5:**
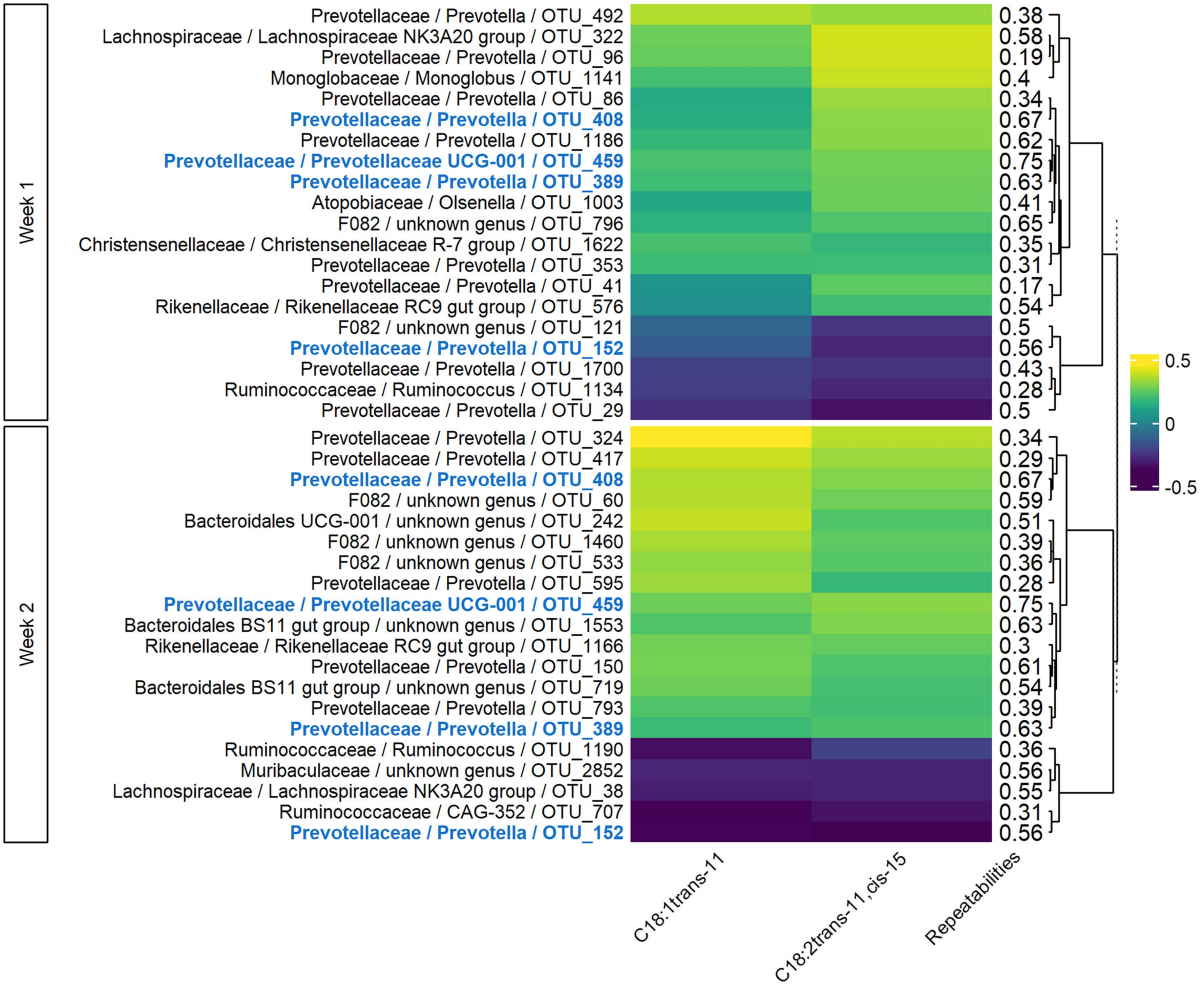
Pearson correlations between bacterial OTUs and milk long-chain fatty acids on both weeks of sampling. Only the 20 more correlated OTUs and the milk LCFAs having at least one significant correlation in both weeks with them were presented. In bold and blue, OTUs conserved from week 1 to week 2.

In both weeks 1 and 2, sPLS models for milk LCFAs included 77 components, with 41 variables retained per component in week 1 and 54 in week 2. For the two main components, the explained variance was 6 and 9% for weeks 1 and 2, respectively. The significant Pearson correlations between the 20 most associated OTUs and C18:1 *trans*-11 and C18:2 *trans*-11, *cis*-15 ranged from −0.39 to −0.22 and from 0.19 to 0.45 (Figure 5). Four OTUs were conserved between week 1 and week 2, two of which were also conserved in the VFA analysis (OTU_408 and OTU_459), and the corresponding correlations were of the same sign. For *Rikenellaceae* and *Ruminococcaceae* families, the sign of the correlation with a given milk LCFA was the same for all the OTUs of the family on both weeks, while bacteria from *Prevotellaceae* family had both positive and negative correlations with the milk LCFAs. Highly repeatable OTUs (>0.50) were particularly selected by the sPLS on both weeks (*p* = 0.04 and *p* < 0.001 for weeks 1 and 2, respectively).

## Discussion

### Biological, Technical, and Computational Effects

*Bacteroidetes*, *Firmicutes*, and *Proteobacteria* were the three main phyla observed in the rumen of adult dairy ewes fed a mixed diet of forages and concentrates, in accordance with that observed in sheep (Belanche et al., 2019; Liu et al., 2020) and cows (Bainbridge et al., 2016; Plaizier et al., 2017). However, the rumen bacterial composition is affected by various factors, mainly biological ones. The most known is the diet composition, often represented by the forage/concentrate ratio that can influence family and genus abundances (Henderson et al., 2015). Another one is the feeding time, influencing diurnal variations in bacterial concentrations in sheep fed once daily (Warner, 1966), with the abundance of families belonging to the phylum Firmicutes increasing with time after feeding (de Assis Lage et al., 2020). In addition, both parity and lactation stage affect bacterial composition by modifying the abundance of phyla and genera (Pitta et al., 2014; Bainbridge et al., 2016; Xue et al., 2018). Although many authors have found some bacterial taxa to be common in all the animals in their study (Jami and Mizrahi, 2012; Xue et al., 2018; Huang et al., 2021), interindividual variations in bacterial composition were observed, and evidence of genetic determinism of abundance of some bacteria has been raised by Sasson et al. (2017) and Difford et al. (2018).

Technical and bioinformatics processing factors are also known to greatly influence the observed bacterial abundance (Pollock et al., 2018; Wang and LêCao, 2020). Diversity and relative abundances vary depending on the type of sampling (de Assis Lage et al., 2020) and DNA extraction methods (Henderson et al., 2013; Gerasimidis et al., 2016). Considering sequencing strategies, the choice of primers influences the estimation of bacterial abundances (Tremblay et al., 2015; Fouhy, 2016) while the sequencing platform influences the read length and error rate (D’Amore et al., 2016; Kchouk et al., 2017). The number of observed OTUs depends on the chosen pipeline, in particular, the clustering and filtering steps (Schloss, 2010). As the sequencing depth is highly variable among the samples, at random or depending on the sequencing run, normalization is usually applied to the data (McMurdie and Holmes, 2014; Weiss et al., 2017).

As the current objective was to determine if the bacterial composition was stable over time, the week was the factor of greatest interest in this study. To allow proper observation, breed, diet, and lactation stages were experimentally controlled. Moreover, the same DNA extraction method was used for the samples of both weeks, only one sequencing run was performed, and the bioinformatic analysis included all samples. The other effects, including genetic lines, SOCS2 genotype, parity, time after feeding, and sequencing depth were corrected through linear models following the methodology used by Martinez Boggio et al. (2021).

### Instability of Bacterial Composition

Assessing the stability of the bacterial composition over a short period of time will support minimizing repeated sampling, as they are invasive for the animals and time-consuming. It is essential to know if the results obtained at one sampling are reliable, whether the conclusions are the same 1 week later. In this study, two criteria were used to assess the stability of the bacterial composition. In the first criterion, bacterial composition was defined as stable when phyla, genera, and OTU proportions were conserved from 1 week to the next for the same animal. In the literature, various methods have been used for assessing bacterial composition stability, such as two one-sided tests (Skarlupka et al., 2019), principal coordinate analysis, and analysis of similarity (Huang et al., 2020; Mamun et al., 2020). In the current study, considering the compositional nature of the data, we used multivariate analyses (working in the Euclidian space), such as sPLS-DA and sPLS. In the second criterion, at the OTU level, the repeatability of the OTUs was used to determine their stability, ranging from 0 (completely unstable) to 1 (completely stable), with OTUs having a repeatability greater than 0.50 being defined as stable. Another method, the Lin’s concordance correlation coefficient, which is also conceptually similar, was used by Zhu et al. (2021) to estimate OTU stability.

Although the week effect was the second largest in the ANOVA, it was significant for only 16% of OTUs. A clustering of the samples by week was observed in the sPLS-DA, mainly characterized by different proportions of *Bacteroidetes* and *Firmicutes*, but less than 6% of the variance was explained. Even if the results of these two analyses suggested bacterial composition instability, the high unexplained variance made them inconclusive. As explained previously, various factors influencing bacterial composition cannot be controlled or corrected for. In our study, such factors impacted the variance explained in the ANOVA and the sPLS-DA analyses. They could be unrecorded technical effects such as (1) contamination from the oral cavity or saliva (Lodge-Ivey et al., 2009; Terré et al., 2012), (2) multiple relocation of the gastric tube in the rumen (Geishauser and Gitzel, 1996), and (3) variable proportions of solid and liquid phases (Henderson et al., 2013; Vaidya et al., 2018); or biological effects such as the stress induced by handling before and during sampling (Yoshihara and Ogawa, 2021).

However, the repeatability of 96% of the OTUs was under 0.5, and it was this large proportion of non-stable OTUs (median repeatability of 0.15) that allowed us to state bacterial composition instability. Similar results were obtained by Jewell et al. (2015); Bainbridge et al. (2016); and Zhu et al. (2021) when working over long periods of 75–122 days, which was expected because of the numerous effects that could alter bacterial composition. The 96% of non-stable OTUs (96%) obtained is consistent with the 97% found by Zhu et al. (2021). Contrasting with our results, some authors obtained stability over short periods, from 3 days to 1 week using analyses comparable to ANOVA and sPLS-DA (Skarlupka et al., 2019; Huang et al., 2020; Mamun et al., 2020). However, they did not consider the compositional nature of the data neither computed the OTU repeatability that demonstrated that most of them were moderately repeatable (<0.50). Moreover, the number of animals used in this study was larger than in the previous ones, allowing for revealing small differences between weeks covered up by uncontrolled alterations of the bacterial composition. Those three specificities of our study allowed to clearly reveal bacterial composition instability over short periods.

### Instability of Correlations With Fatty Acid Phenotypes

The biological links between OTUs and FA phenotypes were compared between week 1 and week 2 to discuss functional ruminal bacterial stability over time. At the OTU level, only three or four OTUs were conserved from week 1 to week 2, with similar correlations to FA phenotypes (Figures 3–5). However, we found that OTUs belonging to a same family presented all correlations of the same sign for a given FA. This phenomenon was observed for some of the most predominant families in the rumen, namely *Lachnospiraceae*, *Rikenellaceae*, and *Ruminococcaceae*, and may suggest functional redundancy (Wohl et al., 2004; Weimer, 2015; Louca et al., 2018). That is to say, different OTUs belonging to the same family may have the same function, allowing them to maintain the organism function despite a variable microbial composition. In addition, OTUs from *Prevotellaceae* family presented both positive and negative correlations with the FAs. The main hypothesis to explain this observation is the large genetic and functional diversity of this family (Stewart et al., 1997; Matsui et al., 2000). Further investigation of these aspects is not possible with 16S rRNA gene sequencing, because as stated by Plummer and Twin (2015), it does not allow access to the species and strain classification necessary to investigate the function of the bacteria identified.

The OTUs generally presented low repeatability, with only 4% of them defined as stable. It is notable that stable OTUs were overrepresented among the OTUs highly correlated with FA phenotypes in both weeks, ranging from 3 to 11 among 20. If the stable OTUs are considered as those having high genetic determinism, it was not surprising to find them highly associated with FA phenotypes, for which a significant part of the variability is related to animal genetics. The correlations between bacteria and FA phenotypes from week 1 to week 2 were not conserved, but OTUs related to FA phenotypes appeared to be the most repeatable.

Week-to-week variation in the rumen bacterial composition of Lacaune dairy ewes was observed, with only 4% of the OTUs being stable, which seemed to alter the correlations between microbiota and FAs phenotypes, as only a few OTUs were conserved between 1 week and the next. Even if FA phenotypes were not linked to the same OTUs from 1 week to the next, it is noticeable that they were particularly associated with stable OTUs, possibly due to common genetic determinism of those traits and OTUs. In conclusion, we proved with a large dataset of 188 dairy ewes that bacterial composition and its phenotypic correlations with fatty acids are not transposable from 1 week to the next. Further work is necessary to confirm those results, as very few studies were performed on microbiota stability over short periods of time, and none using compositional data approach. We hope that our study will be a first step in identifying microbiota members with high repeatability and thus potential heritability, to then consider the possibility of genetic selection.

## Data Availability Statement

The datasets presented in this study can be found in online repositories. The names of the repository/repositories and accession number(s) can be found at: https://www.ncbi.nlm.nih.gov/, PRJNA765197.

## Ethics Statement

The animal study was reviewed and approved by the appropriate Ethical Committee (APAFIS#6292-2016080214271984 v8). Written informed consent was obtained from the owners for the participation of their animals in this study.

## Author Contributions

CM-E and AM conceived the experiments and participated in sample collection and processing. GMB performed data processing. SF performed statistical analyses under the supervision of GMB and CM-E. SF, CM-E, GMB, and AM participated in the interpretation of the results. SF wrote the manuscript. All authors contributed to the article and approved the submitted version.

## Funding

The experiment was funded by Génétique Animale and Physiologie Animale et Systèmes d’Elevage divisions of INRAE.

## Conflict of Interest

The authors declare that the research was conducted in the absence of any commercial or financial relationships that could be construed as a potential conflict of interest.

## Publisher’s Note

All claims expressed in this article are solely those of the authors and do not necessarily represent those of their affiliated organizations, or those of the publisher, the editors and the reviewers. Any product that may be evaluated in this article, or claim that may be made by its manufacturer, is not guaranteed or endorsed by the publisher.

## Footnotes

^1^ https://www.r-project.org/
