# Supplementary material for: Variation in Rumen Bacteria of Lacaune Dairy Ewes From One Week to the Next
Source: Front Microbiol. 2022 Jun 23;13:848518. doi: 10.3389/fmicb.2022.848518 (PMC9260014; doi:10.3389/fmicb.2022.848518)
Supplement: Supplementary file 1 [file Data_Sheet_1.docx]

Supplementary Material

**Supplementary Table 1.**

Taxonomy, loadings values, repeatability, abundance and percentage of zeros for the 57 OTUs selected on the component 1 of the sPLS-DA.

| OTUs | Loadings | Phylum | Genus | Association | Repeatability | Abundance | Percentage of zeros |
| --- | --- | --- | --- | --- | --- | --- | --- |
| OTU_5 | -0.124 | Firmicutes | Acetitomaculum | Week 1 | 0.58 | 37,817 | 0 |
| OTU_34 | -0.099 | Bacteroidetes | Prevotella | Week 1 | 0.54 | 6,364 | 0.9 |
| OTU_279 | -0.135 | Bacteroidetes | Prevotella | Week 1 | 0.51 | 6,307 | 1.8 |
| OTU_586 | 0.099 | Bacteroidetes | unknown genus | Week 2 | 0.48 | 871 | 12.6 |
| OTU_311 | 0.082 | Bacteroidetes | unknown genus | Week 2 | 0.47 | 1,575 | 15.8 |
| OTU_766 | -0.005 | Bacteroidetes | Prevotellaceae YAB2003 group | Week 1 | 0.45 | 1,044 | 20.7 |
| OTU_294 | 0.179 | Bacteroidetes | Rikenellaceae RC9 gut group | Week 2 | 0.41 | 2,427 | 5.4 |
| OTU_1141 | 0.423 | Firmicutes | Monoglobus | Week 2 | 0.40 | 515 | 28.8 |
| OTU_73 | -0.051 | Firmicutes | NK4A214 group | Week 1 | 0.38 | 3,465 | 0.5 |
| OTU_1380 | -0.036 | Bacteroidetes | Prevotella | Week 1 | 0.37 | 215 | 57.7 |
| OTU_507 | 0.051 | Bacteroidetes | Rikenellaceae RC9 gut group | Week 2 | 0.36 | 1,528 | 6.8 |
| OTU_328 | -0.006 | Bacteroidetes | Prevotellaceae NK3B31 group | Week 1 | 0.34 | 1,150 | 10.4 |
| OTU_2261 | -0.005 | Spirochaetota | Treponema | Week 1 | 0.30 | 237 | 52.7 |
| OTU_534 | -0.111 | Spirochaetota | Treponema | Week 1 | 0.30 | 1,216 | 19.8 |
| OTU_483 | 0.172 | Bacteroidetes | Rikenellaceae RC9 gut group | Week 2 | 0.29 | 1,614 | 9.5 |
| OTU_529 | 0.010 | Bacteroidetes | unknown genus | Week 2 | 0.29 | 565 | 34.7 |
| OTU_601 | 0.092 | Bacteroidetes | Rikenellaceae RC9 gut group | Week 2 | 0.29 | 720 | 18.9 |
| OTU_1464 | 0.134 | Fibrobacterota | Fibrobacter | Week 2 | 0.28 | 245 | 43.2 |
| OTU_95 | -0.029 | Bacteroidetes | unknown genus | Week 1 | 0.26 | 5,236 | 0 |
| OTU_530 | 0.082 | Bacteroidetes | Prevotella | Week 2 | 0.25 | 1,576 | 6.8 |
| OTU_874 | -0.212 | Spirochaetota | Treponema | Week 1 | 0.24 | 956 | 11.3 |
| OTU_636 | 0.039 | Bacteroidetes | Prevotella | Week 2 | 0.23 | 515 | 30.6 |
| OTU_1390 | -0.209 | Firmicutes | Ruminococcus | Week 1 | 0.23 | 191 | 54.1 |
| OTU_1393 | -0.089 | Firmicutes | NK4A214 group | Week 1 | 0.22 | 398 | 32.0 |
| OTU_472 | 0.042 | Bacteroidetes | unknown genus | Week 2 | 0.22 | 1,944 | 5.0 |
| OTU_4 | 0.147 | Bacteroidetes | unknown genus | Week 2 | 0.22 | 25,680 | 0 |
| OTU_40 | -0.289 | Firmicutes | Pseudobutyrivibrio | Week 1 | 0.21 | 5,386 | 0 |
| OTU_1067 | 0.056 | Firmicutes | unknown genus | Week 2 | 0.21 | 369 | 42.8 |
| OTU_956 | -0.056 | Firmicutes | unknown genus | Week 1 | 0.21 | 557 | 24.8 |
| OTU_43 | 0.123 | Bacteroidetes | Prevotella | Week 2 | 0.20 | 5,454 | 0 |
| OTU_673 | 0.083 | Proteobacteria | Sutterella | Week 2 | 0.19 | 713 | 23.0 |
| OTU_191 | 0.057 | Bacteroidetes | Prevotella | Week 2 | 0.18 | 3,443 | 0.9 |
| OTU_1086 | -0.034 | Spirochaetota | Treponema | Week 1 | 0.17 | 873 | 11.3 |
| OTU_10 | -0.028 | Firmicutes | Acetitomaculum | Week 1 | 0.16 | 14,862 | 0 |
| OTU_45 | 0.123 | Bacteroidetes | Prevotella | Week 2 | 0.15 | 6109 | 1.4 |
| OTU_2 | 0.135 | Bacteroidetes | unknown genus | Week 2 | 0.14 | 46,230 | 0 |
| OTU_402 | 0.151 | Fibrobacterota | Fibrobacter | Week 2 | 0.14 | 1,826 | 6.3 |
| OTU_1265 | 0.024 | Firmicutes | unknown genus | Week 2 | 0.13 | 271 | 53.6 |
| OTU_774 | -0.005 | Firmicutes | Christensenellaceae R-7 group | Week 1 | 0.13 | 456 | 27.5 |
| OTU_1459 | 0.146 | Bacteroidetes | Prevotella | Week 2 | 0.11 | 209 | 55.9 |
| OTU_1074 | -0.076 | Firmicutes | NK4A214 group | Week 1 | 0.11 | 821 | 9.9 |
| OTU_57 | -0.096 | Bacteroidetes | unknown genus | Week 1 | 0.10 | 9,137 | 0 |
| OTU_873 | -0.197 | Spirochaetota | Treponema | Week 1 | 0.09 | 1,007 | 13.1 |
| OTU_136 | 0.175 | Bacteroidetes | Prevotella | Week 2 | 0.09 | 4,817 | 1.8 |
| OTU_312 | 0.163 | Bacteroidetes | Prevotella | Week 2 | 0.08 | 1,617 | 7.2 |
| OTU_52 | -0.037 | Bacteroidetes | Prevotella | Week 1 | 0.06 | 8,464 | 0.5 |
| OTU_250 | 0.265 | Fibrobacterota | Fibrobacter | Week 2 | 0.05 | 4,774 | 3.2 |
| OTU_1854 | 0.196 | Firmicutes | unknown genus | Week 2 | 0.03 | 341 | 45.5 |
| OTU_305 | 0.029 | Bacteroidetes | Prevotella | Week 2 | 0.03 | 2,517 | 5.0 |
| OTU_39 | 0.260 | Bacteroidetes | Prevotellaceae UCG-003 | Week 2 | 0.03 | 5,644 | 0.9 |
| OTU_1543 | -0.011 | Firmicutes | Acetitomaculum | Week 1 | 0.01 | 256 | 45.5 |
| OTU_30 | 0.041 | Bacteroidetes | unknown genus | Week 2 | 0.01 | 6,806 | 1.4 |
| OTU_1188 | -0.018 | Firmicutes | Acetitomaculum | Week 1 | *NA* | 305 | 43.2 |
| OTU_61 | 0.068 | Bacteroidetes | Prevotella | Week 2 | *NA* | 9,255 | 1.4 |
| OTU_610 | 0.086 | Bacteroidetes | unknown genus | Week 2 | *NA* | 637 | 23.0 |
| OTU_717 | -0.087 | Firmicutes | Pseudobutyrivibrio | Week 1 | *NA* | 796 | 16.2 |
| OTU_76 | 0.135 | Bacteroidetes | unknown genus | Week 2 | *NA* | 4,851 | 0.9 |

**Supplementary Table 2.**

Taxonomy, loadings values, repeatability, abundance and percentage of zeros for the 57 OTUs selected on the component 2 of the sPLS-DA.

| Clusters | Loadings | Phylum | Genus | Association | Repeatability | Abundance | Percentage_zeros |
| --- | --- | --- | --- | --- | --- | --- | --- |
| Cluster_279 | 0.165 | Bacteroidetes | Prevotella | Week 1 | 0.51 | 6307 | 1.8 |
| Cluster_46 | 0.087 | Bacteroidetes | Prevotella | Week 1 | 0.47 | 5341 | 0.5 |
| Cluster_611 | 0.043 | Bacteroidetes | Prevotella | Week 1 | 0.41 | 763 | 32.4 |
| Cluster_166 | 0.072 | Firmicutes | V9D2013 group | Week 1 | 0.41 | 1994 | 4.5 |
| Cluster_86 | 0.019 | Bacteroidetes | Prevotella | Week 2 | 0.34 | 3406 | 3.2 |
| Cluster_513 | -0.026 | Bacteroidetes | unknown genus | Week 2 | 0.34 | 1032 | 11.3 |
| Cluster_18 | 0.083 | Bacteroidetes | Prevotella | Week 2 | 0.33 | 10026 | 0.5 |
| Cluster_81 | 0.099 | Bacteroidetes | Prevotella | Week 1 | 0.32 | 3674 | 0.9 |
| Cluster_133 | 0.341 | Bacteroidetes | Prevotella | Week 1 | 0.32 | 6317 | 0.5 |
| Cluster_171 | 0.091 | Bacteroidetes | unknown genus | Week 1 | 0.29 | 3210 | 0.9 |
| Cluster_413 | 0.016 | Bacteroidetes | Prevotella | Week 1 | 0.29 | 2120 | 5.9 |
| Cluster_127 | -0.003 | Bacteroidetes | Prevotella | Week 2 | 0.27 | 2420 | 1.4 |
| Cluster_1355 | -0.029 | Firmicutes | Mogibacterium | Week 2 | 0.27 | 302 | 42.8 |
| Cluster_6 | 0.227 | Bacteroidetes | Prevotella | Week 1 | 0.27 | 17095 | 0.5 |
| Cluster_154 | 0.186 | Bacteroidetes | Prevotella | Week 1 | 0.25 | 3375 | 2.3 |
| Cluster_306 | 0.067 | Firmicutes | Lachnospiraceae UCG-008 | Week 1 | 0.25 | 1082 | 8.1 |
| Cluster_698 | 0.053 | Bacteroidetes | Prevotella | Week 1 | 0.24 | 675 | 22.5 |
| Cluster_2303 | 0.068 | Bacteroidetes | Prevotella | Week 1 | 0.21 | 329 | 40.5 |
| Cluster_77 | 0.021 | Proteobacteria | Succinivibrionaceae UCG-002 | Week 1 | 0.20 | 6306 | 4.1 |
| Cluster_96 | 0.027 | Bacteroidetes | Prevotella | Week 1 | 0.19 | 3050 | 2.3 |
| Cluster_13 | 0.016 | Bacteroidetes | Prevotella | Week 2 | 0.18 | 19450 | 1.4 |
| Cluster_94 | 0.027 | Proteobacteria | Succinivibrionaceae UCG-002 | Week 1 | 0.17 | 3966 | 2.7 |
| Cluster_41 | 0.415 | Bacteroidetes | Prevotella | Week 1 | 0.17 | 21315 | 0 |
| Cluster_16 | -0.026 | Firmicutes | Lachnospiraceae NK3A20 group | Week 1 | 0.16 | 21892 | 0 |
| Cluster_93 | 0.178 | Bacteroidetes | Prevotella | Week 1 | 0.16 | 3052 | 2.7 |
| Cluster_467 | 0.094 | Spirochaetota | Treponema | Week 1 | 0.13 | 1272 | 14.0 |
| Cluster_770 | -0.010 | Bacteroidetes | Prevotella | Week 2 | 0.13 | 427 | 32.4 |
| Cluster_814 | -0.137 | Firmicutes | Family XIII AD3011 group | Week 2 | 0.13 | 404 | 35.1 |
| Cluster_2250 | -0.079 | Bacteroidetes | unknown genus | Week 2 | 0.09 | 226 | 46.8 |
| Cluster_136 | 0.001 | Bacteroidetes | Prevotella | Week 2 | 0.09 | 4817 | 1.8 |
| Cluster_1917 | -0.014 | Firmicutes | Acetitomaculum | Week 2 | 0.08 | 206 | 64.4 |
| Cluster_1672 | -0.020 | Proteobacteria | Suttonella | Week 2 | 0.08 | 519 | 29.3 |
| Cluster_69 | 0.078 | Bacteroidetes | Prevotella | Week 2 | 0.08 | 3976 | 0.5 |
| Cluster_59 | 0.154 | Bacteroidetes | Prevotella | Week 2 | 0.07 | 13489 | 1.4 |
| Cluster_400 | 0.024 | Bacteroidetes | Prevotella | Week 1 | 0.07 | 895 | 15.8 |
| Cluster_25 | 0.294 | Bacteroidetes | Prevotella | Week 2 | 0.07 | 8603 | 0.9 |
| Cluster_1410 | -0.190 | Firmicutes | Christensenellaceae R-7 group | Week 2 | 0.07 | 197 | 53.6 |
| Cluster_52 | 0.404 | Bacteroidetes | Prevotella | Week 1 | 0.06 | 8464 | 0.5 |
| Cluster_63 | 0.143 | Bacteroidetes | Prevotella | Week 2 | 0.05 | 2621 | 2.7 |
| Cluster_200 | 0.070 | Bacteroidetes | Prevotella | Week 1 | 0.03 | 1722 | 5.4 |
| Cluster_26 | 0.149 | Bacteroidetes | Prevotella | Week 1 | 0.03 | 6965 | 0.9 |
| Cluster_7 | 0.100 | Bacteroidetes | unknown genus | Week 2 | 0.02 | 14832 | 0 |
| Cluster_159 | -0.024 | Firmicutes | [Ruminococcus] gauvreauii group | Week 1 | 0.02 | 2481 | 0.5 |
| Cluster_650 | 0.025 | Firmicutes | Lachnospiraceae UCG-008 | Week 1 | 0.01 | 511 | 27.9 |
| Cluster_359 | 0.098 | Bacteroidetes | unknown genus | Week 1 | 0.01 | 1072 | 12.2 |
| Cluster_1050 | -0.053 | Firmicutes | Christensenellaceae R-7 group | Week 2 | *NA* | 312 | 37.8 |
| Cluster_1062 | -0.001 | Firmicutes | Family XIII AD3011 group | Week 2 | *NA* | 918 | 10.4 |
| Cluster_126 | 0.143 | Bacteroidetes | Prevotella | Week 1 | *NA* | 2977 | 1.8 |
| Cluster_1440 | -0.133 | Firmicutes | Marvinbryantia | Week 2 | *NA* | 455 | 27.0 |
| Cluster_146 | 0.068 | Fibrobacteria | Fibrobacter | Week 2 | *NA* | 9101 | 1.4 |
| Cluster_17 | 0.138 | Bacteroidetes | Prevotella | Week 2 | *NA* | 9234 | 0.9 |
| Cluster_251 | 0.093 | Bacteroidetes | Prevotella | Week 1 | *NA* | 1673 | 2.3 |
| Cluster_428 | -0.032 | Firmicutes | Lachnospiraceae NK3A20 group | Week 1 | *NA* | 608 | 15.3 |
| Cluster_451 | -0.015 | Firmicutes | Colidextribacter | Week 2 | *NA* | 729 | 13.1 |
| Cluster_598 | 0.065 | Bacteroidetes | unknown genus | Week 1 | *NA* | 713 | 39.2 |
| Cluster_61 | 0.025 | Bacteroidetes | Prevotella | Week 2 | *NA* | 9255 | 1.4 |
| Cluster_75 | 0.105 | Bacteroidetes | Prevotella | Week 1 | *NA* | 3637 | 1.4 |
